# Supplementary figures and images for: Annexin A6 mediates calcium-dependent exosome secretion during plasma membrane repair
Source: eLife. 2023 May 19;12:e86556. doi: 10.7554/eLife.86556 (PMC10241516; doi:10.7554/eLife.86556)

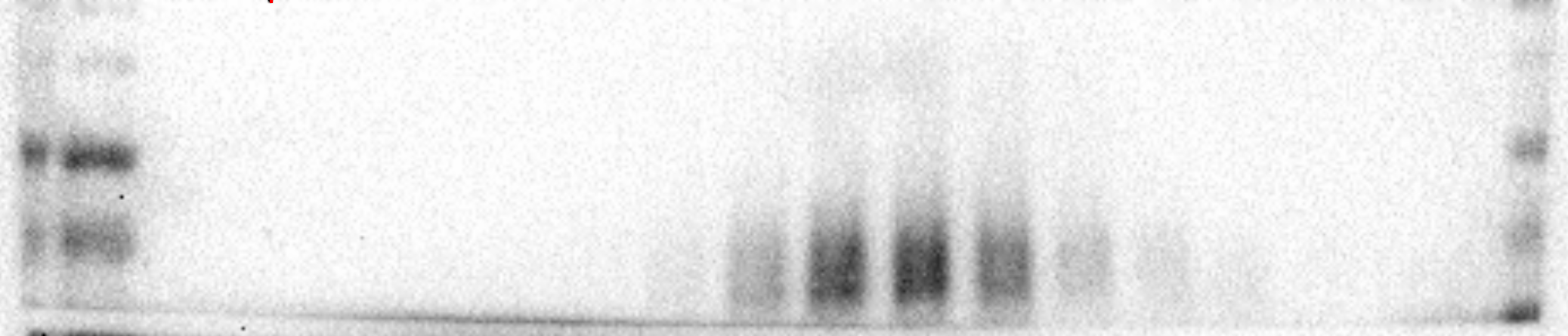

Supplement: Figure 2—source data 1. [file elife-86556-fig2-data1.zip › Source Data - Figure 2/Figure 2D_CD63-DMSO.tif]

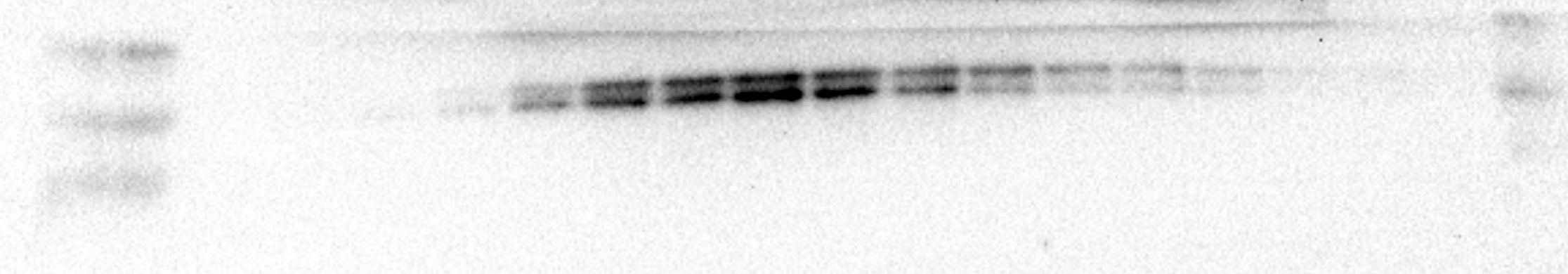

Supplement: Figure 2—source data 1. [file elife-86556-fig2-data1.zip › Source Data - Figure 2/Figure 2D_ANXA2-Ionomycin.tif]

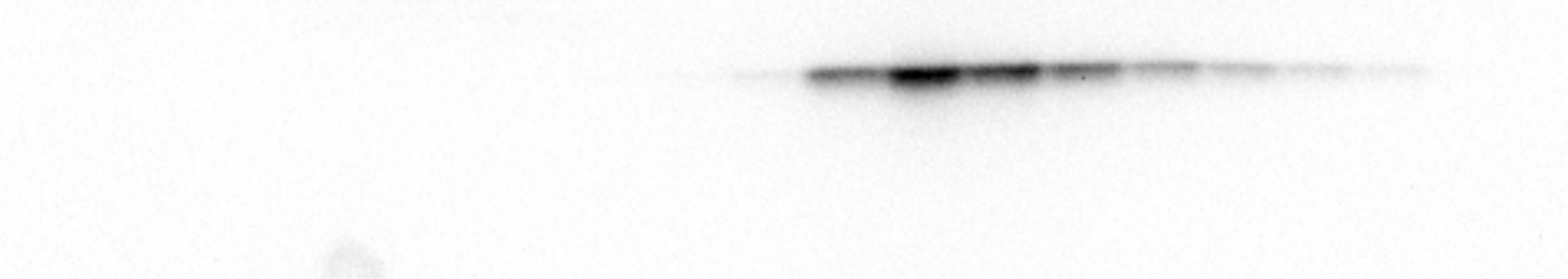

Supplement: Figure 2—source data 1. [file elife-86556-fig2-data1.zip › Source Data - Figure 2/Figure 2D_CD9-Ionomycin.tif]

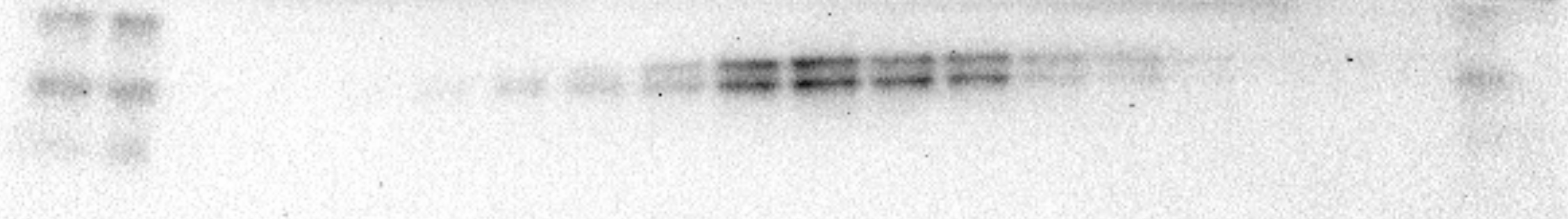

Supplement: Figure 2—source data 1. [file elife-86556-fig2-data1.zip › Source Data - Figure 2/Figure 2D_ANXA2-DMSO.tif]

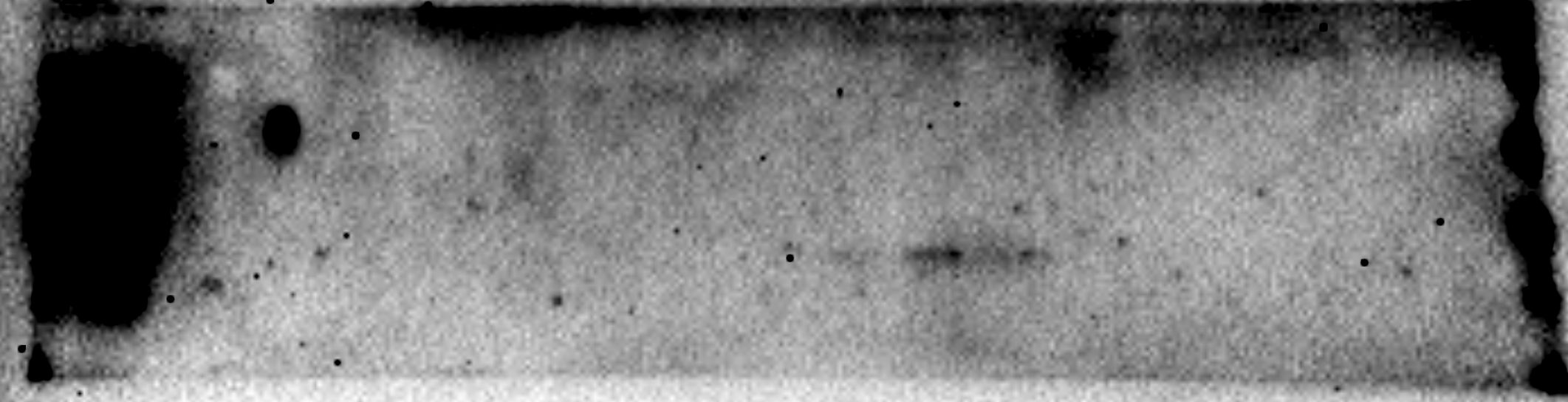

Supplement: Figure 2—source data 1. [file elife-86556-fig2-data1.zip › Source Data - Figure 2/Figure 2D_TSG101-Ionomycin.tif]

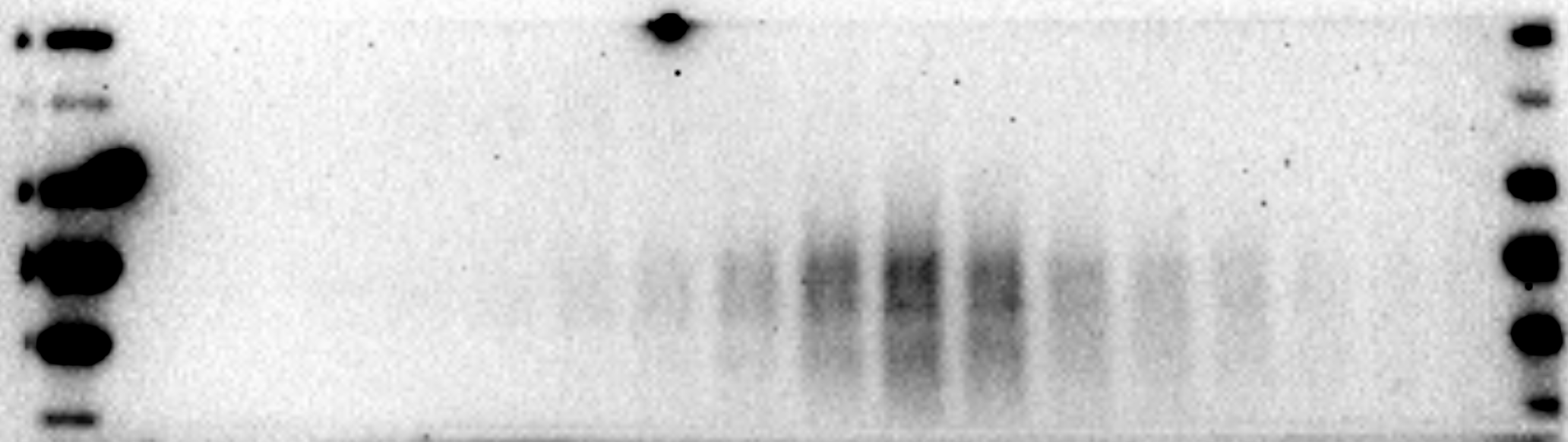

Supplement: Figure 2—source data 1. [file elife-86556-fig2-data1.zip › Source Data - Figure 2/Figure 2D_CD63-Ionomycin.tif]

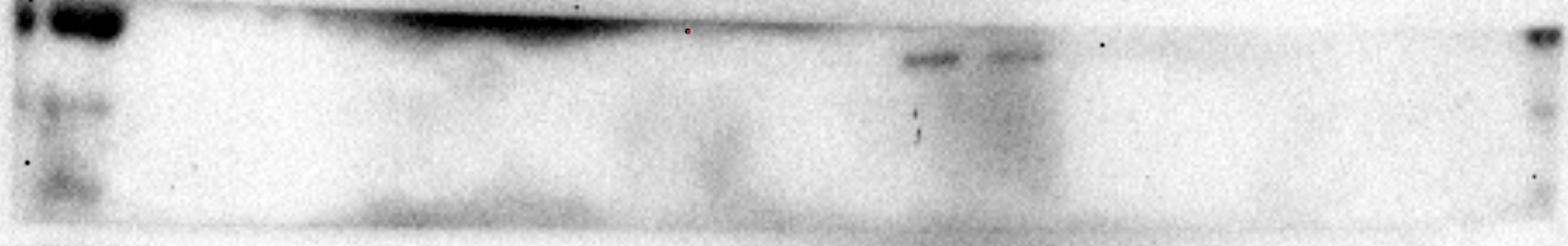

Supplement: Figure 2—source data 1. [file elife-86556-fig2-data1.zip › Source Data - Figure 2/Figure 2D_TSG101-DMSO.tif]

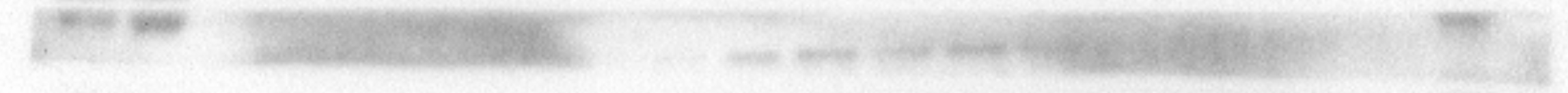

Supplement: Figure 2—source data 1. [file elife-86556-fig2-data1.zip › Source Data - Figure 2/Figure 2D_Flot2-DMSO.tif]

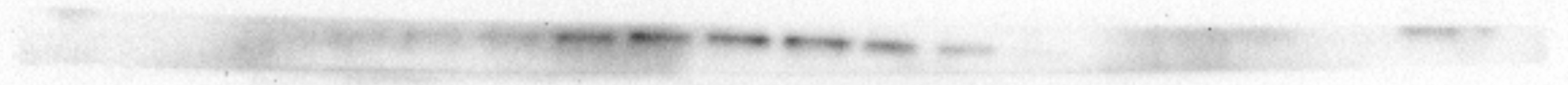

Supplement: Figure 2—source data 1. [file elife-86556-fig2-data1.zip › Source Data - Figure 2/Figure 2D_Flot2-Ionomycin.tif]

Figure 2 - Source Data

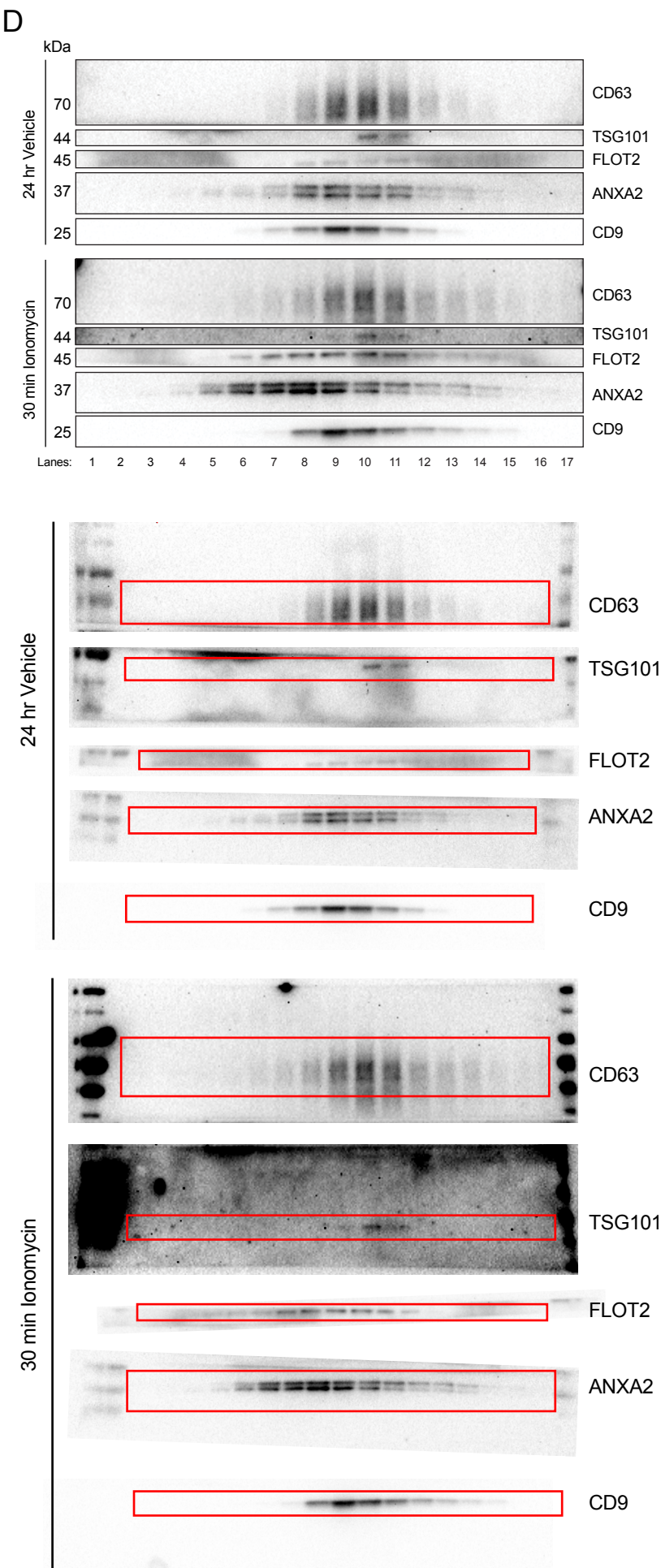

Supplement: Figure 2—source data 1. [file elife-86556-fig2-data1.zip › Source Data - Figure 2/Figure 2 - Source Data.pdf]

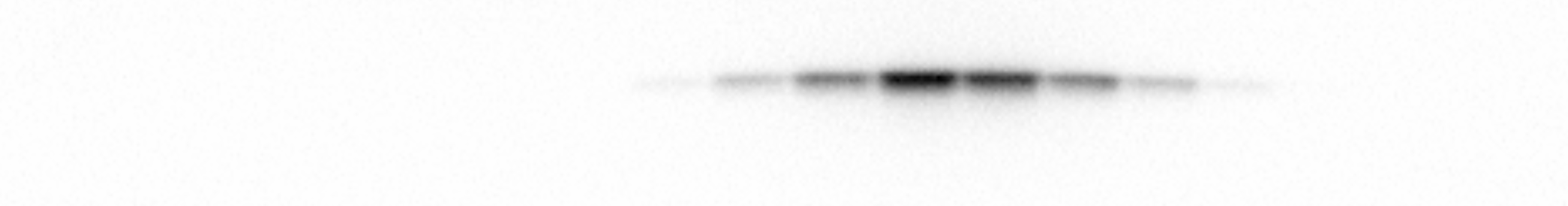

Supplement: Figure 2—source data 1. [file elife-86556-fig2-data1.zip › Source Data - Figure 2/Figure 2D_CD9-DMSO.tif]

Figure 2 - Figure supplement 1 - Source Data

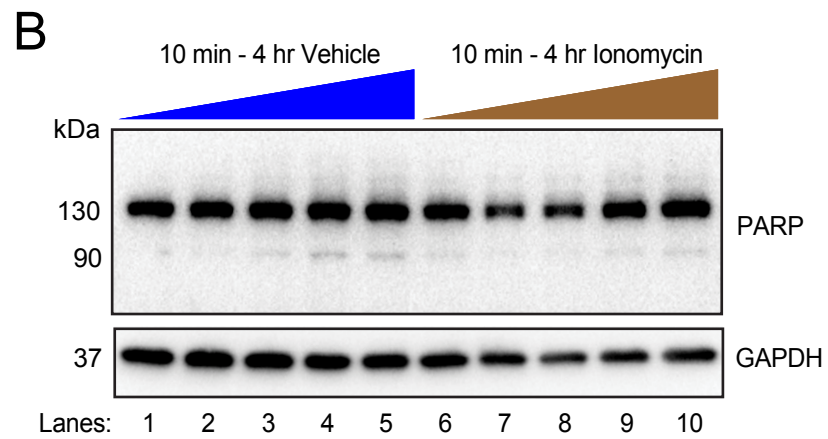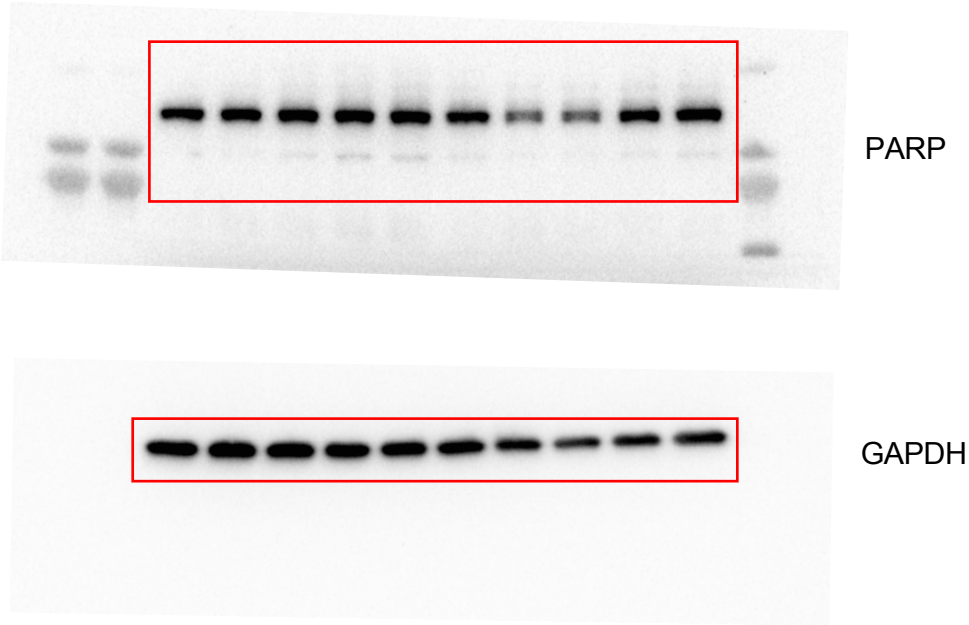

Supplement: Figure 2—figure supplement 1—source data 1. [file elife-86556-fig2-figsupp1-data1.zip › Source Data - Figure 2 - Figure supplement 1/Figure 2 - Figure supplement 1 - Source Data.pdf]

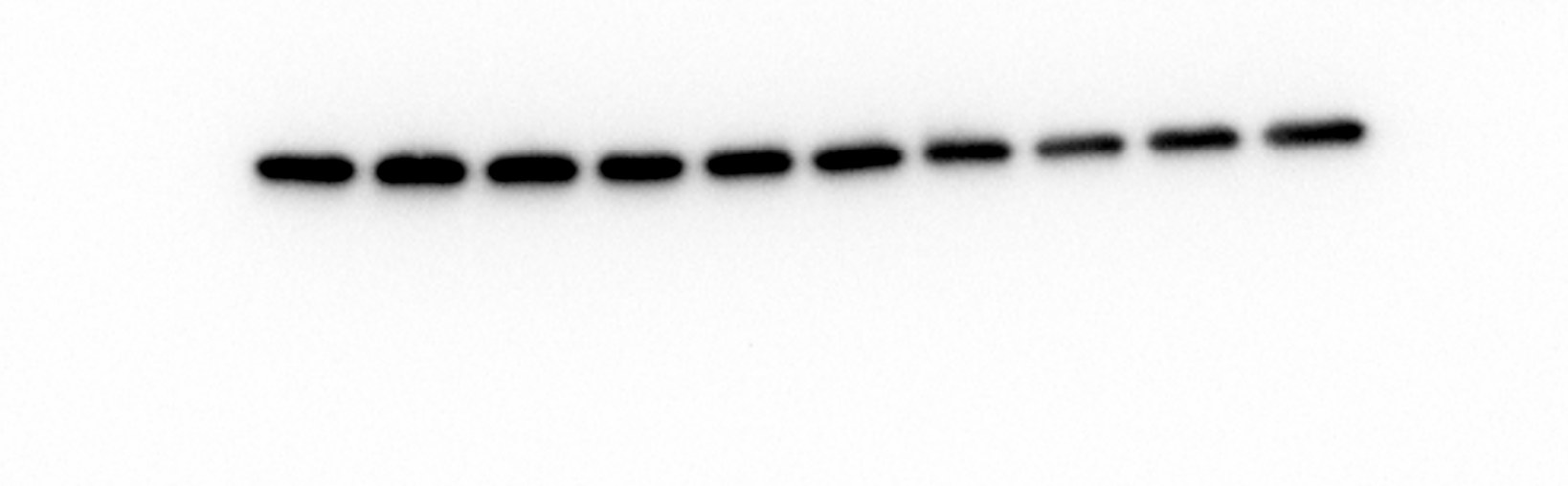

Supplement: Figure 2—figure supplement 1—source data 1. [file elife-86556-fig2-figsupp1-data1.zip › Source Data - Figure 2 - Figure supplement 1/Figure 2 - Figure supplement 1B_GAPDH.tif]

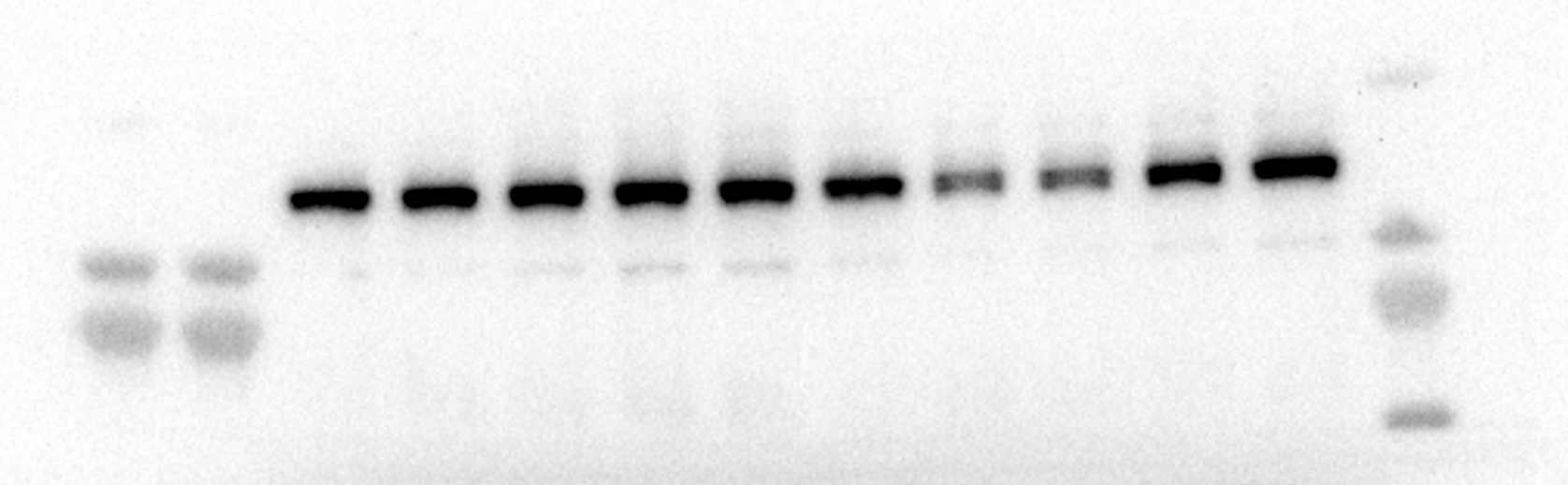

Supplement: Figure 2—figure supplement 1—source data 1. [file elife-86556-fig2-figsupp1-data1.zip › Source Data - Figure 2 - Figure supplement 1/Figure 2 - Figure supplement 1B_PARP.tif]

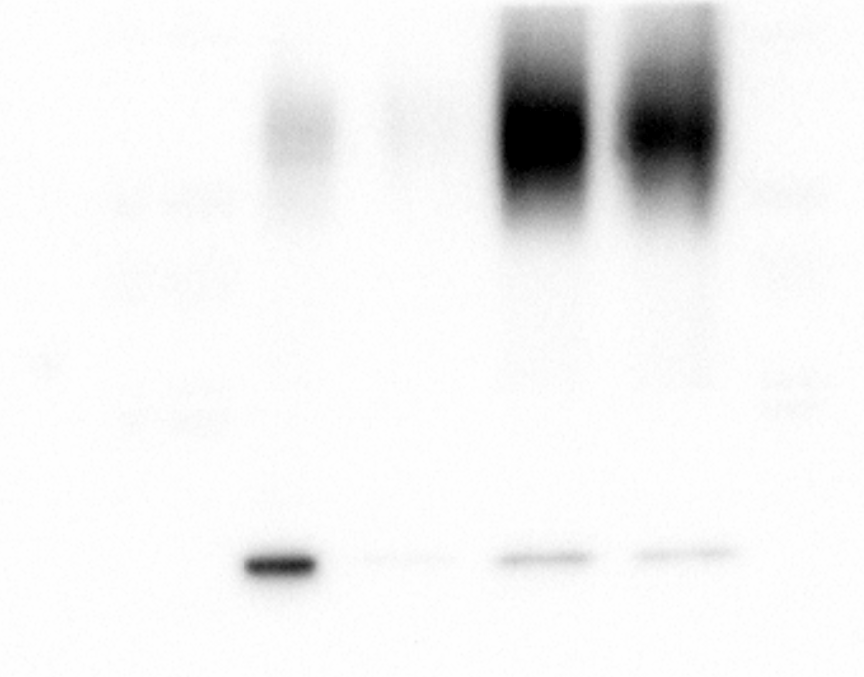

Supplement: Figure 3—source data 1. [file elife-86556-fig3-data1.zip › Source Data - Figure 3/Figure 3B_LAMP1(Top) and GAPDH(Bottom).tif]

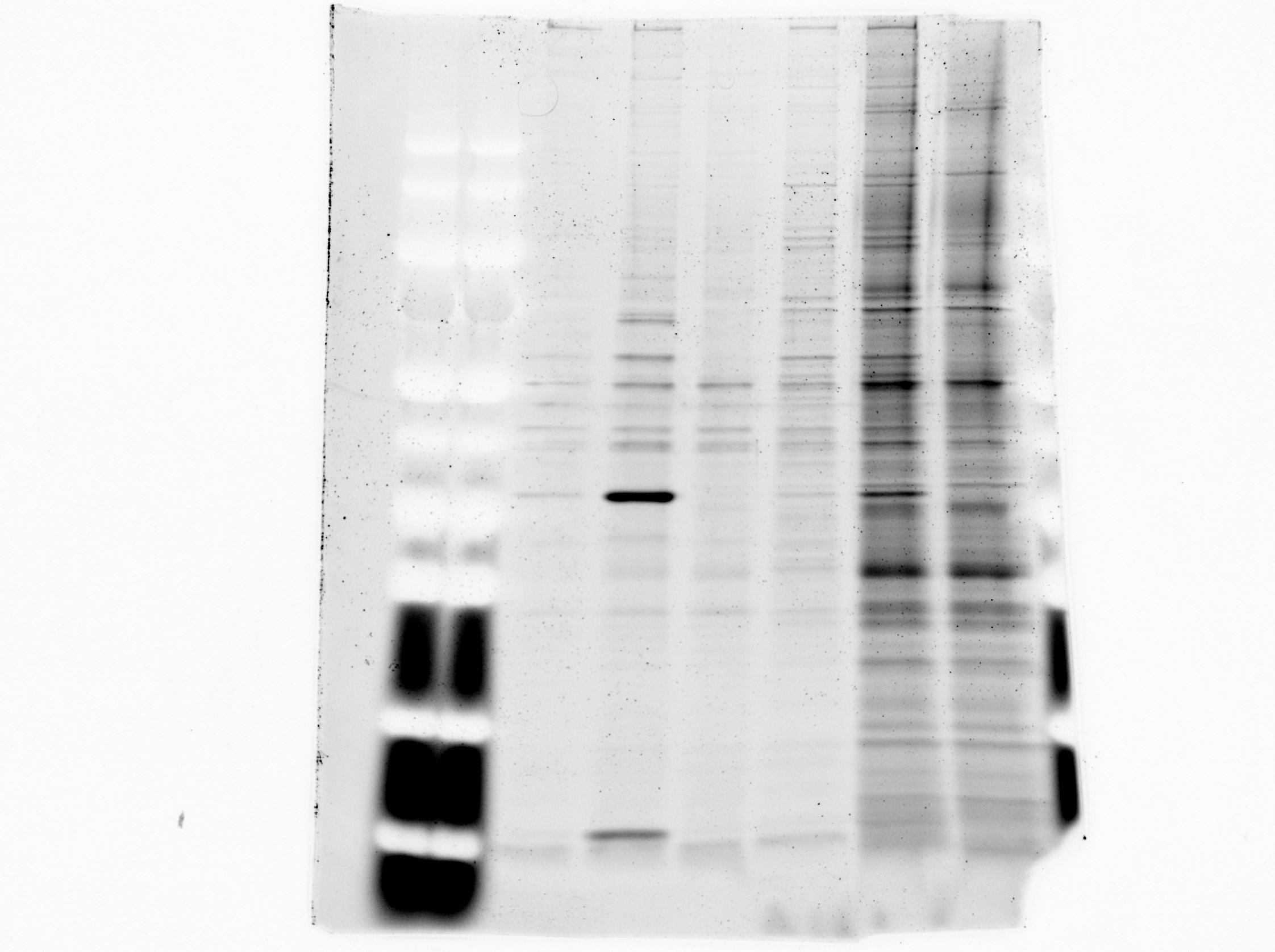

Supplement: Figure 3—source data 1. [file elife-86556-fig3-data1.zip › Source Data - Figure 3/Figure 3C_Sypro Ruby.tif]

Figure 3 - Source Data 1

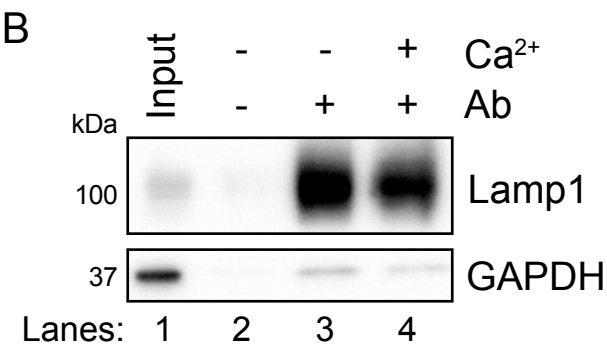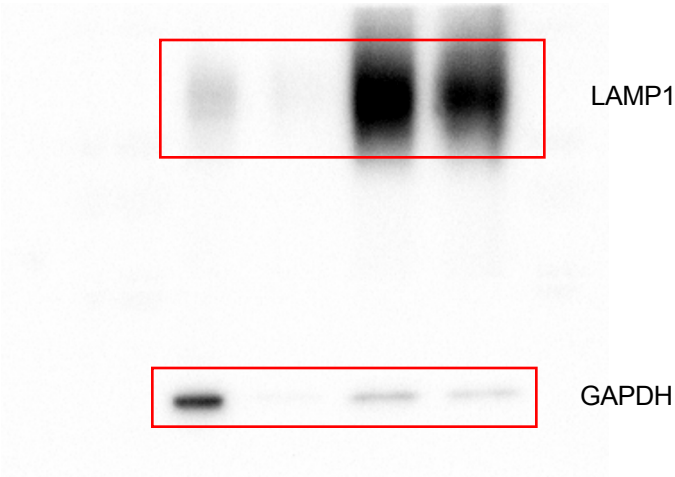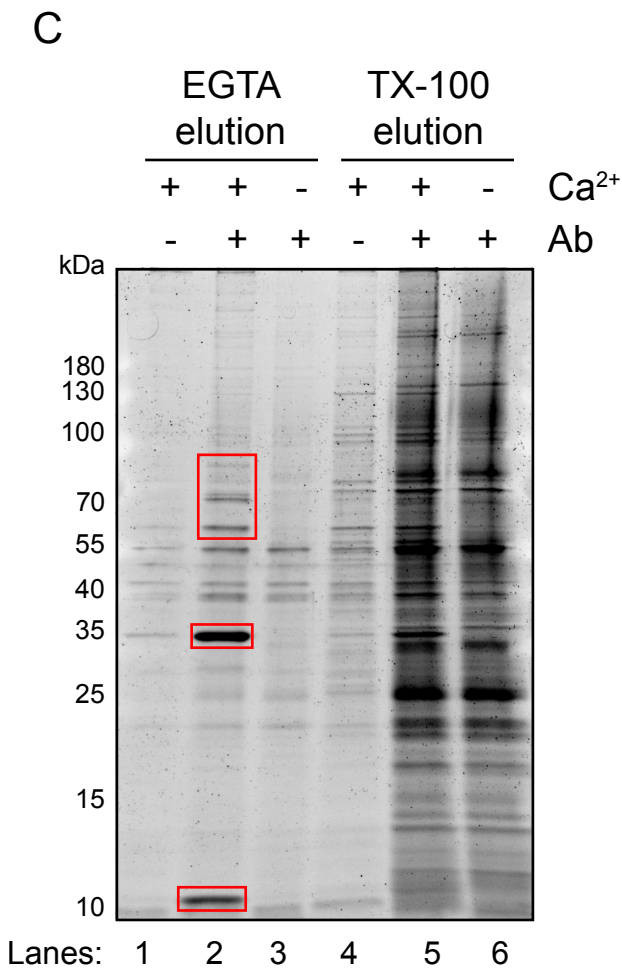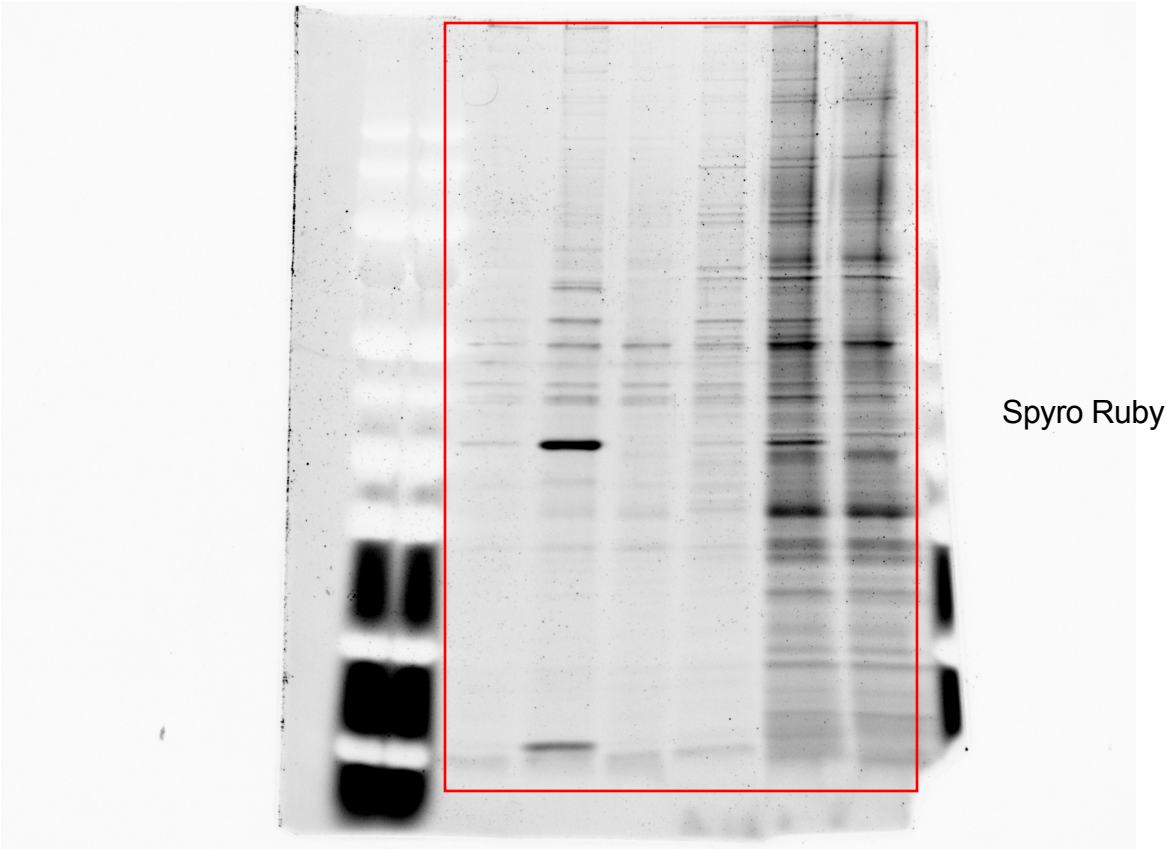

Supplement: Figure 3—source data 1. [file elife-86556-fig3-data1.zip › Source Data - Figure 3/Figure 3 - Source Data 1.pdf]

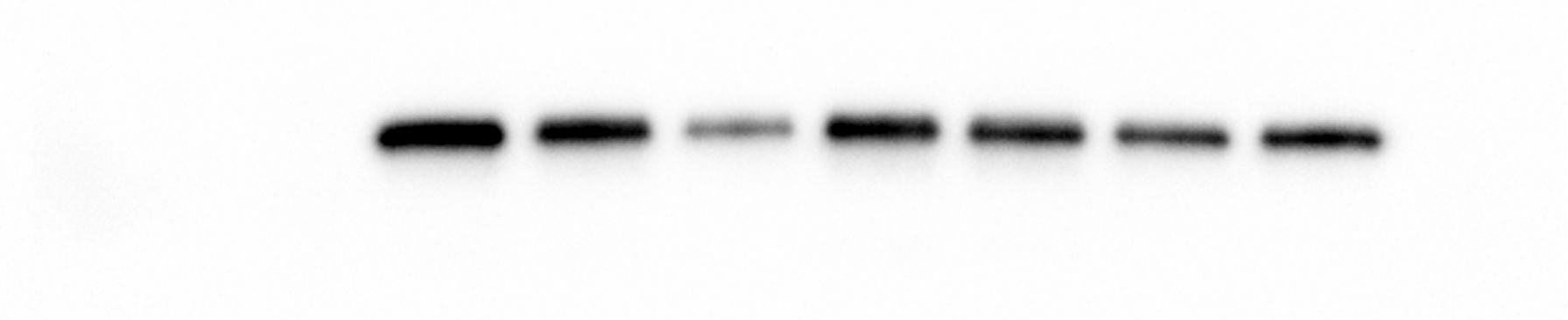

Supplement: Figure 4—source data 1. [file elife-86556-fig4-data1.zip › Source Data - Figure 4/Figure 4B_GAPDH.tif]

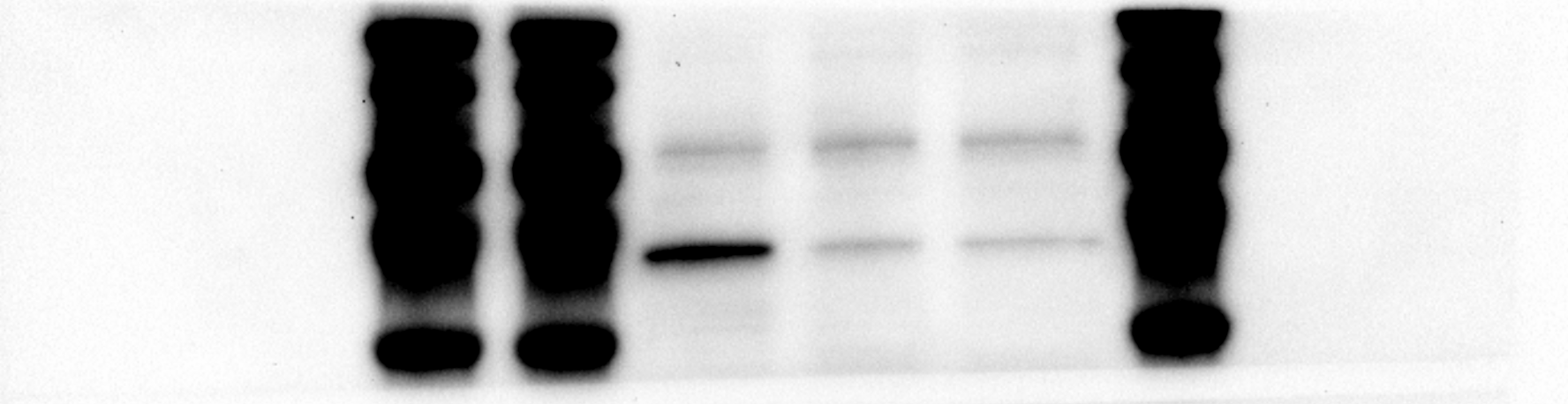

Supplement: Figure 4—source data 1. [file elife-86556-fig4-data1.zip › Source Data - Figure 4/Figure 4A_ANXA6.tif]

Figure 4 - Source Data

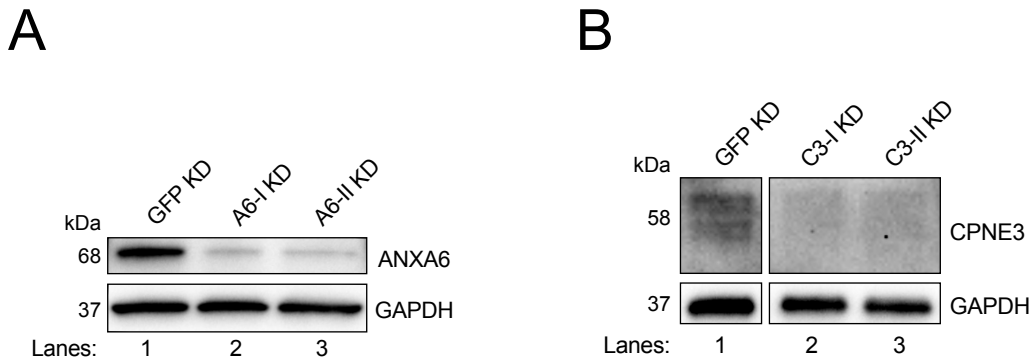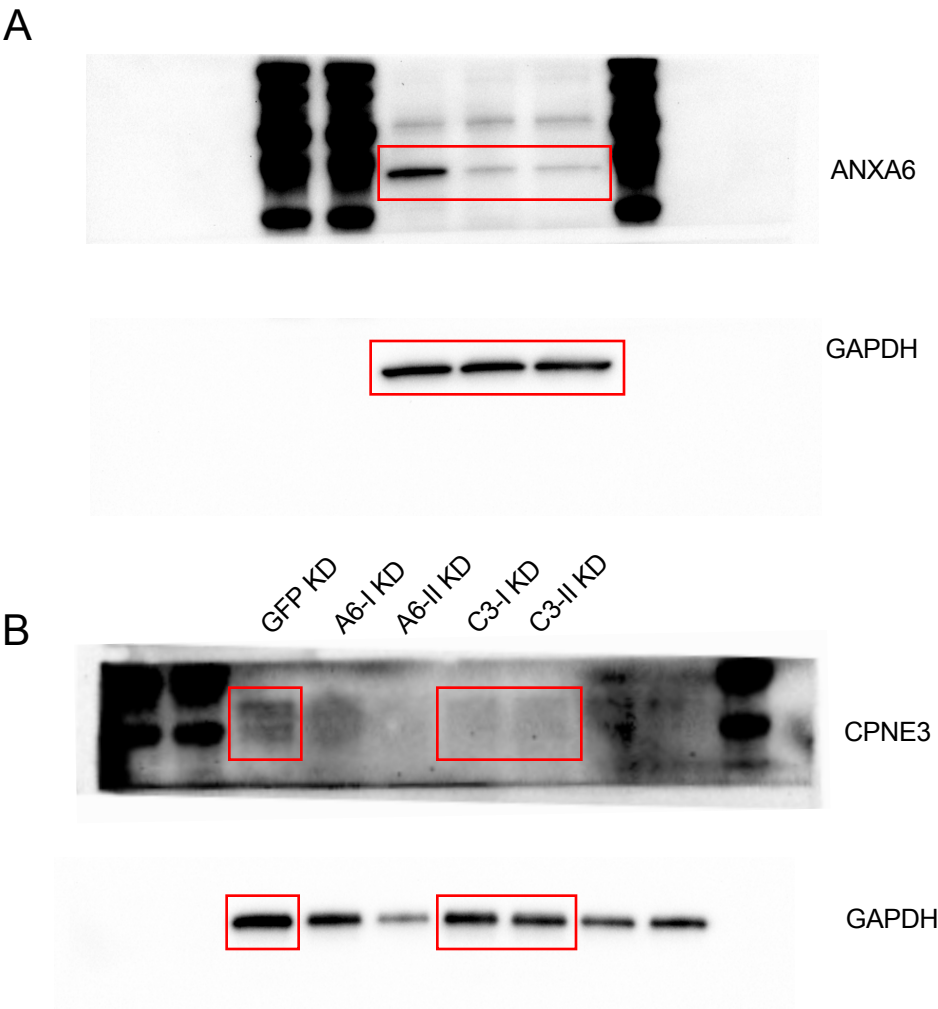

Supplement: Figure 4—source data 1. [file elife-86556-fig4-data1.zip › Source Data - Figure 4/Figure 4 - Source Data.pdf]

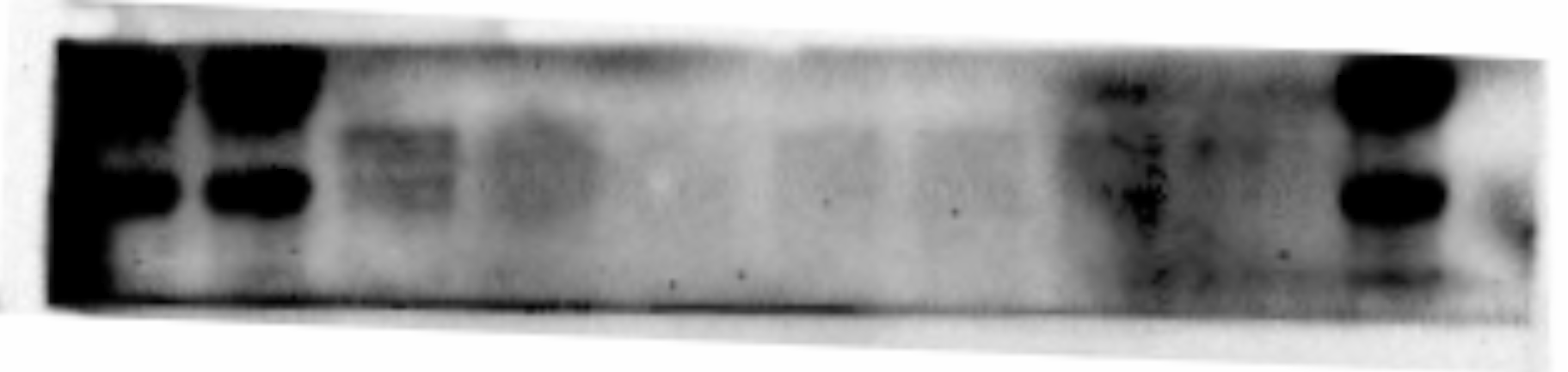

Supplement: Figure 4—source data 1. [file elife-86556-fig4-data1.zip › Source Data - Figure 4/Figure 4B_CPNE3.tif]

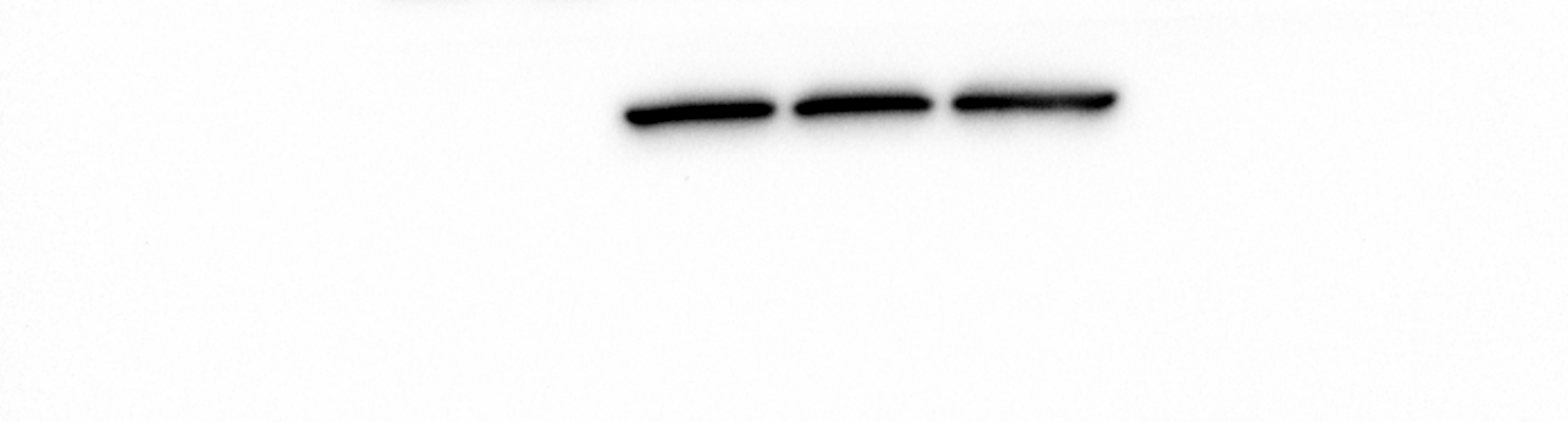

Supplement: Figure 4—source data 1. [file elife-86556-fig4-data1.zip › Source Data - Figure 4/Figure 4A_GAPDH.tif]

Figure 4 - Figure supplement 1 - Source Data

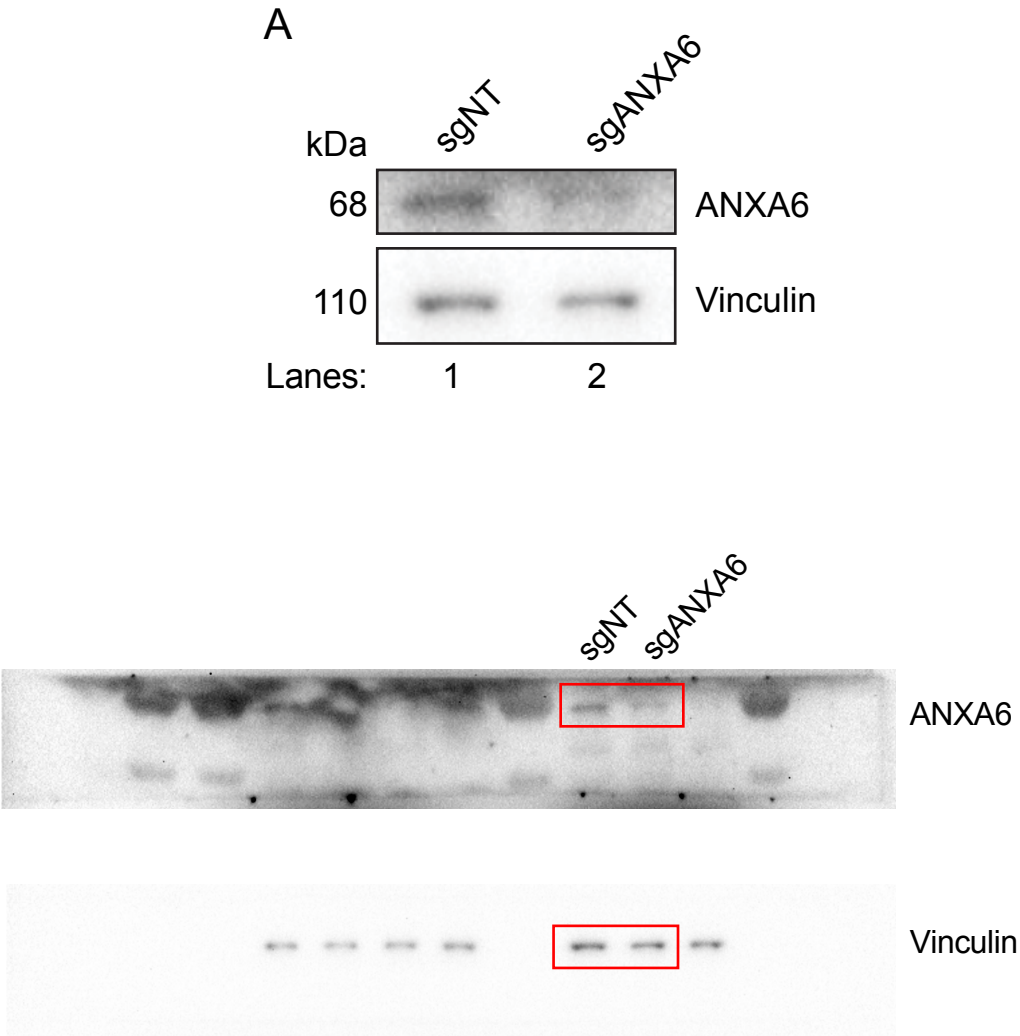

Supplement: Figure 4—figure supplement 1—source data 1. [file elife-86556-fig4-figsupp1-data1.zip › Source Data - Figure 4 - Figure supplement 1/Figure 4 - Figure supplement 1 - Source Data.pdf]

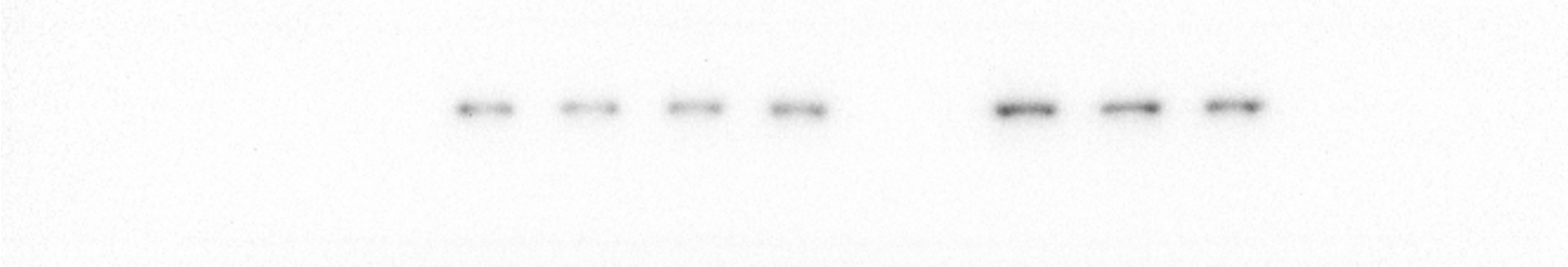

Supplement: Figure 4—figure supplement 1—source data 1. [file elife-86556-fig4-figsupp1-data1.zip › Source Data - Figure 4 - Figure supplement 1/Figure 4 - Figure supplement 1A_Vinculin.tif]

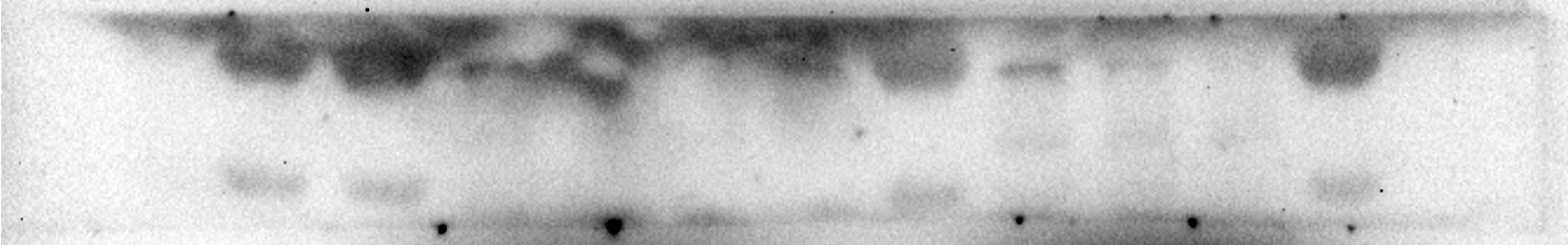

Supplement: Figure 4—figure supplement 1—source data 1. [file elife-86556-fig4-figsupp1-data1.zip › Source Data - Figure 4 - Figure supplement 1/Figure 4 - Figure supplement 1A_ANXA6.tif]
